# Conformal uncertainty quantification to evaluate predictive fairness of foundation AI model for skin lesion classes across patient demographics

**DOI:** 10.1007/s13755-025-00412-z

**Published:** 2025-12-24

**Authors:** Swarnava Bhattacharyya, Umapada Pal, Tapabrata Chakraborti

**Affiliations:** 1 Indian Statistical Institute, Kolkata, India; 2 The Alan Turing Institute and University College London, London, UK

**Keywords:** Algorithmic fairness, Vision transformer (ViT), Foundation models, Skin lesion classification, Conformal prediction, Uncertainty quantification, Transparent trustworthy AI, Class imbalance

## Abstract

Deep learning based diagnostic AI systems based on medical images are starting to provide similar performance as human experts. However, these data-hungry complex systems are inherently black boxes and therefore slow to be adopted for high-risk applications like healthcare. This problem of lack of transparency is exacerbated in the case of recent large foundation models, which are trained in a self-supervised manner on millions of data points to provide robust generalisation across a range of downstream tasks. The embeddings generated from them happen through a process that is not interpretable, and hence not easily trustable for clinical applications. To address this timely issue, we deploy conformal analysis to quantify the predictive uncertainty of a vision transformer (ViT)-based foundation model across patient demographics with respect to sex, age, and ethnicity for the task of skin lesion classification using several public benchmark datasets. The significant advantage of this method is that conformal analysis is method independent, and it not only provides a coverage guarantee at the population level but also provides an uncertainty score for each individual. This is used to demonstrate the effectiveness of utilizing these embeddings for specialized tasks like diagnostic classification, meanwhile reducing computational costs. Secondly, the public benchmark datasets we used had severe class imbalance in terms of the number of samples in different classes. We used a model-agnostic dynamic F1-score-based sampling during model training, which helped to stabilize the class imbalance. We investigate the effects on uncertainty quantification (UQ) with or without this bias mitigation step. Thus, our results show how this can be used as a fairness metric to evaluate the robustness of the feature embeddings of the foundation model (Google DermFoundation), advancing the trustworthiness and fairness of clinical AI.

## Introduction

Skin cancer remains a significant global health concern, with melanoma accounting for more than 5% of the total cancer cases diagnosed in the US, causing more than 8000 deaths in 2024, with multiple non-melanoma cancer subtypes having largely unreported incidence counts in millions every year,^1^ [1]. With over 8430 people estimated to die from melanoma in 2025 in the USA alone [2], figures from around the world highlight its escalating incidence. Among these, basal cell carcinoma (BCC) and squamous cell carcinoma (SCC) are the two most common forms, with over 5.9 million and 1.8 million cases recorded in 2017, respectively [1], limited locally to the region of primary occurrence[3]. Meanwhile, melanoma is the most serious type of skin cancer due to its propensity for metastasis, with 75% of deaths associated with skin cancer being caused by melanoma [3]. Geographical variability is also noteworthy. As an example, regions such as Australia and New Zealand report high incidence rates, with opportunistic early detection being the major treatment method [4]. Furthermore, with more people diagnosed with skin cancer in the US each year than all other cancers combined, the urgency for continued research in skin cancer classification, prevention, and treatment has never been more critical [2].

The integration of artificial intelligence (AI) in dermatological practice has emerged as a transformative approach for skin cancer diagnosis. Modern deep learning based diagnostic systems have demonstrated performance levels comparable to human experts [5]. Due to the data-hungry nature of deep learning models, using additional metadata during training often helps in increasing the performance of such models significantly [6] and others. The field of deep learning based medical image analysis is currently seeing a shift from convolutional neural networks (CNNs) towards large vision transformer ViT-based models [7]. These models are popularly referred to as foundation models as they are often trained in a general-purpose self-supervised manner over millions of images to generate rich feature embeddings, which can then be fed into a task-specific bespoke model. Khoiwal et al. [8] have shown how using specialized pre-trained foundation models can surpass traditionally trained and tested classifiers with much lesser resource requirements. These foundation models output vector embeddings, which are feature-rich, lower-dimensional data generated from the original datasets, which can significantly improve AI model performance while reducing resource requirements. Restrepo et al. [9] have showcased the usage of embeddings in low-resource settings for medical tasks, which show no performance drop, along with considerable resource optimizations. General-purpose pre-trained foundation models can work equally well for a large variety of subtasks, especially in fields such as medical AI [10]. However, all of these existing approaches share some drawbacks, both from the model and data perspectives. State-of-the-art deep learning models are complex (CNNs have millions of trainable parameters, while large ViTs may have billions) and hence inherently opaque to interpretation. But for such models to be adopted in high-risk applications like healthcare, it is crucial to overcome this clinical translational bottleneck through decision transparency. On the other hand, it is important to leverage the power of these state-of-the-art foundation models, leading to a dichotomy. The second constraint is related to the quality and quantity of data availability. There is a severe class imbalance problem persisting in most available healthcare datasets — a well-known long-tail problem in computer vision [11]. However, in certain medical imaging tasks, there can be additional bias across patient demographics with respect to sex, age, or race[12]. For example, in the case of skin cancer, there is a significant majority of Caucasian patients, and thus the model might have higher predictive accuracy for those patients, leading to a lack of algorithmic fairness.

Our work addresses both of the above challenges, that is, predictive trustworthiness and fairness in skin lesion classification. Firstly, we do not shy away from using cutting-edge ViT-based foundation models to achieve state-of-the-art performance (we use the Google Derm Foundation model [13]), but rather demonstrate the robustness and trustworthiness of the model by rigorously quantifying the predictive uncertainty of the AI pipeline using conformal prediction-based uncertainty quantification. Conformal prediction is a calibration-based model-agnostic statistical method for quantifying uncertainty of predictive models with a coverage guarantee and has great potential in high-risk applications such as medical AI [14–17]. Along with uncertainty quantification in classification and prediction models, conformal prediction can be used for generative [18, 19] and personalised [20] healthcare solutions, which showcase the dynamism of the process. Using a hold-out calibration set of samples, conformal analysis provides a marginal coverage guarantee that the set of predicted labels in the test phase (called the conformal set) will contain the true label at a user-specified level of significance. In addition, it provides a confidence bound for each patient, thus increasing the trustworthiness of the system. To address the bias of class imbalance between patient ethnicities, we introduce a new dynamic custom sampler F1 between training epochs and an ensemble learning-based strategy on both sets of data with Caucasian and Asian patients. This resulted in an increase in the robustness of predictive performance between both ethnic groups of patients, which was quantified with accuracy metrics and conformal uncertainty quantification. Although our work focuses on skin lesion classification, both approaches (F1-based dynamic sampling and conformal prediction) are model-agnostic and task-agnostic statistical approaches and thus can be used as a generalized framework for measuring algorithmic fairness. Thus, the main contributions of this paper are:

- Demonstrated generalisation abilities of pre-trained foundation model embeddings for skin cancer classification across target datasets without any retraining or fine-tuning.
- Introduced a novel F1-weight sampler for reducing class imbalance in dermatological datasets.
- Implemented uncertainty quantification using conformal prediction across protected attributes like gender, age, and ethnicity for algorithmic fairness evaluation.
- Showcased the usefulness of the conformal prediction pipeline using metrics and graphical figures for health AI purposes

The rest of the paper is organised as follows. Section Methodology presents the proposed methodology, including pseudo-codes. Section Experimental setup described the experimental setup, including the description of the datasets used. Section Results includes detailed results with respect to classification performance as well as algorithmic fairness. The paper is concluded in Sect. Conclusion.

## Methodology

**Fig. 1:**
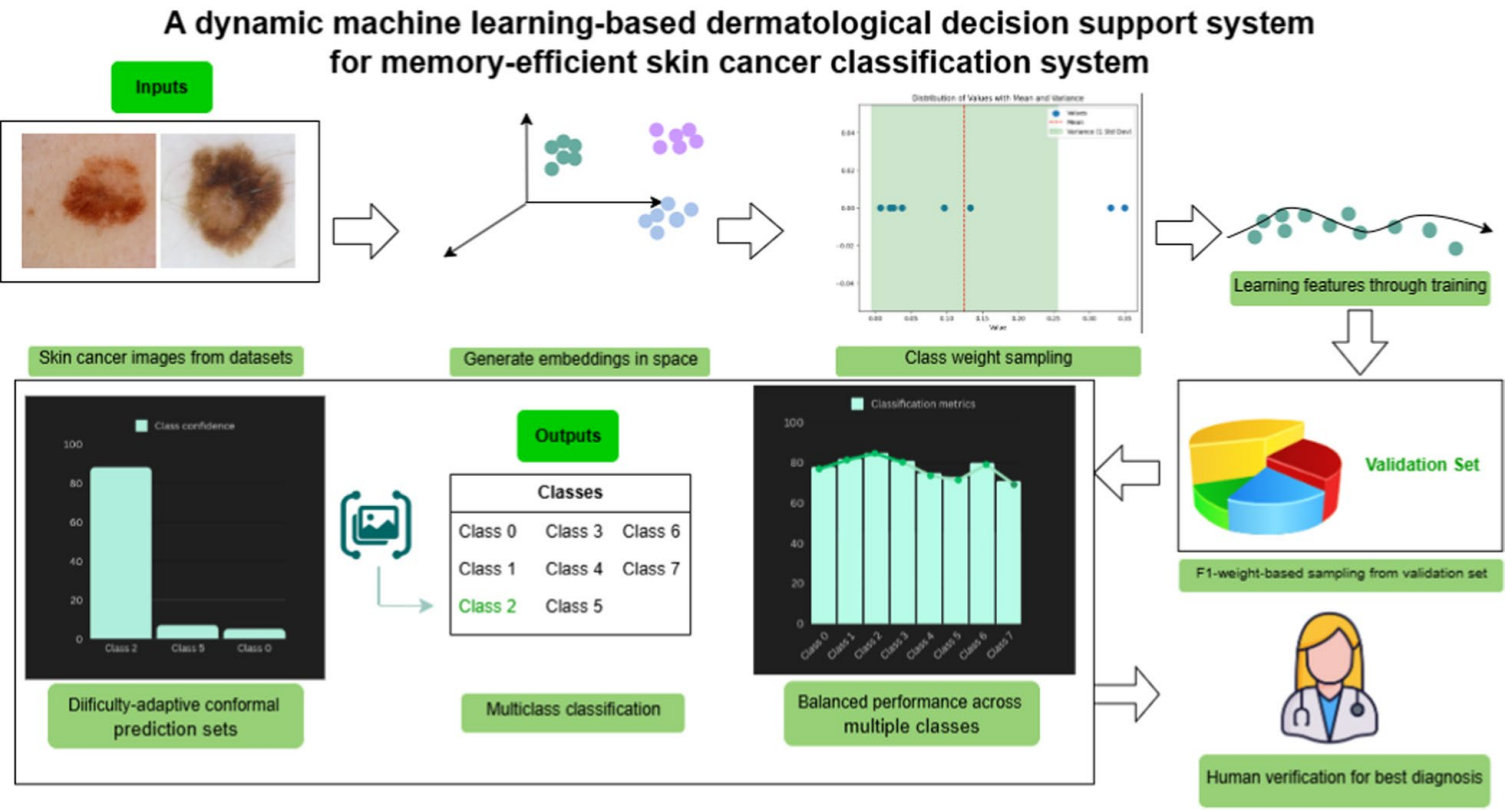
Pipeline of the proposed system. Our AI-driven workflow can be integrated with skin cancer diagnosis systems for supporting manual diagnosis of patients by classifying skin cancer from dermatological images, especially in lower time and memory constraints. In real-time, it can classify image samples into skin cancer subtypes and produce prediction sets showing the guarantee associated with the most-probable predictions

Our methodology for the skin lesion classification process and subsequent conformal uncertainty prediction is an approach that combines the power of state-of-the-art foundation models with the trustworthiness of conformal prediction-based uncertainty quantification, as discussed in this Section (Fig. 1).

### F1-weight-based dynamic sampler

Since both datasets are heavily class-imbalanced, with maximum class frequencies being several times bigger than some of the minority classes, we built a dynamic, model-agnostic, epoch-wise sampling algorithm based on F1-score-based weights for classes, greatly balancing the classwise performance. Two important parameters for our custom sampler were the *threshold value* $\lambda$ and the *minimum weight* $\beta$, and the sampler update rule involving them is described in Algorithm 1 below. The threshold decides the cutoff for which classes will be baseline sampled and which will be F1-weight sampled, thereby maintaining a healthy balance between minority and majority classes. The minimum weight is the weight value by which the majority classes are baseline sampled. The choice of using F1-sampling over other balancing techniques is mainly due to its effectiveness and adaptability. Using existing resources like the validation samples, the model can effectively adjust itself periodically during the training process and focus its learning more towards classes whose samples it is finding more challenging to classify. This balances the training schedules inherently without any external inputs. However, readjusting itself after every epoch of training might lead to overfitting the training data. This is where the adaptability of this mechanism shines — we can deploy it as required in the training pipeline based on our dataset distribution and model performance.

**Algorithm 1:**
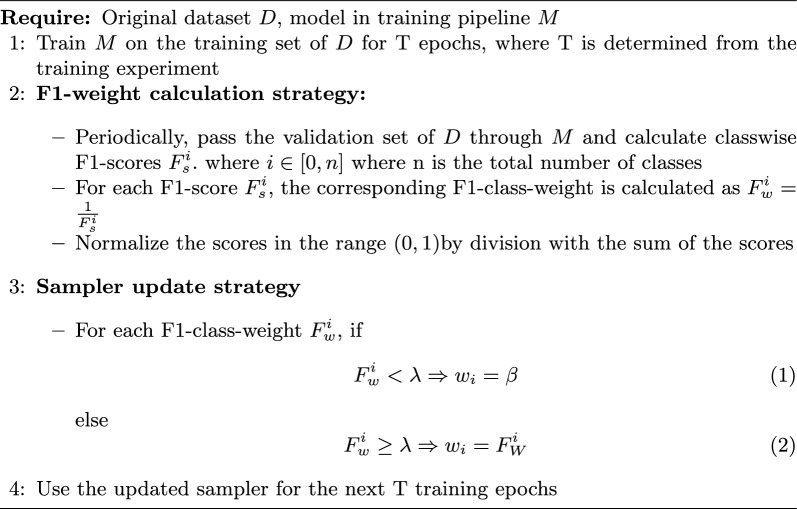
Challenge-regulated F1-score sampling for highly imbalanced datasets

### AI model architectures

We have used a state-of-the-art vision transformer-based foundation model (Google DermFoundation model) for feature embedding. The advantage of using this model is that it has been specifically pre-trained on extracting robust embeddings from dermatology images; hence, these embeddings can be used directly without the need for fine-tuning. This enables us to use a relatively simple multi-layer perception (MLP) type neural network as the classifier head. Since the MLP network has only a few hidden layers, the training overhead gets substantially reduced as the foundation model backbone remains frozen. This keeps the model lightweight. Hence, it is suitable for deployment in clinical settings that are constrained in computational resources, while preserving the power of the foundation model. For the MLP models, we used a neural network with 2048 input neurons, 6 blocks each consisting of a fully connected layer with half the neurons from the previous block, a 1D batch normalization layer, an activation layer, and a dropout layer, followed by a final fully connected layer after the last block. This architecture proved effective with the embeddings for classification, as it correctly learned the feature representations in the embeddings. Since the embeddings generated from the two datasets were fundamentally different due to the difference in dermatological features within images, for the combined training approach, we used the Balanced Random Forest, which aggregated a large number of weak learners to produce a strong outcome based on ensemble learning. This tactic proves effective in tackling the covariate shift present in the joint distribution of the two datasets.

### Conformal prediction for uncertainty quantification

Conformal prediction is a rigorous statistical calibration technique for uncertainty quantification of predictive models [21]. At the user-defined level of significance, it provides a marginal guarantee that the true prediction will be contained in the predicted set of output labels for classification or the predicted range for regression tasks [22]. Additionally, for each individual (that is, the test sample), it provides an uncertainty bound of prediction, which is useful for personalised healthcare or precision medicine. This increases the trustworthiness of the AI predictions, and the healthcare providers can make a more informed and interpretable decision based on the conformal prediction.

**Fig. 2:**
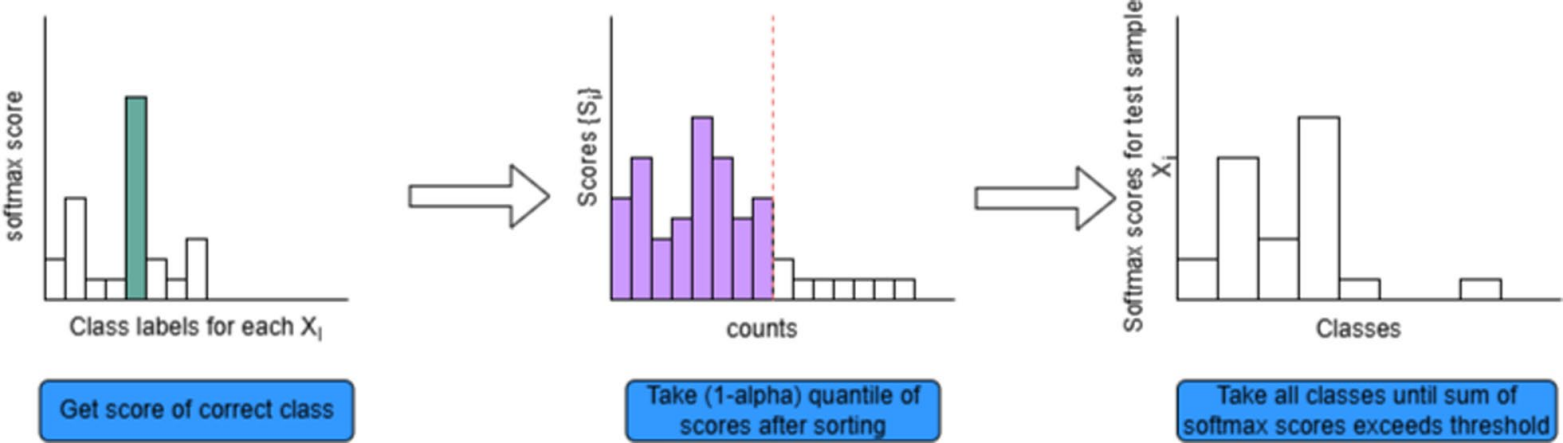
Detailed steps in the conformal prediction set generation process

For our work, the steps to generate the conformal prediction sets were as follows. We build a separate calibration set consisting of a small number of samples (approx. 500), which the model has never encountered during training or testing. We use the deviation between predicted and true labels to calculate nonconformity scores for the calibration samples, which help to define the threshold for confidence intervals. We sort the nonconformity scores and take the $1-\alpha$ quantiles with some finite correction as the threshold score for generating conformal prediction sets, where $\alpha$ is the level of significance for the coverage guarantee (Fig. 2).

3 $$\begin{aligned} 1-\alpha \le \mathbb {P}\bigl (Y_{\text {test}} \in C(X_{\text {test}})\bigr )\le 1-\alpha +\frac{1}{n+1} \end{aligned}$$

where $\bigl (X_\text {test}, Y_\text {test})$ is a test set point from the same distribution as the calibration set, and $\alpha \in [0,1]$ is the user-chosen error rate, and *n* is the size of the calibration set. For test samples with which the model faces considerable difficulty, the number of labels in the prediction set increases—so as to say, the length of the prediction set for any test sample is indicative of the challenge faced by the model while classifying it.

## Experimental setup

We have used two public benchmark skin lesion classification datasets for our work: the ISIC 2019 challenge dataset and the ASAN skin cancer dataset. To maintain an unbiased approach, we applied a fixed set of preprocessing transformations to images from both datasets. Each image is resized to 64x64 pixels. Random transformations, that is, horizontal and vertical flipping, rotating by 90 degrees, and transposition, were employed. We adjusted the brightness and contrast of the transformed images within a set limit of 0.8 and 1.2 of the original values. The images obtained from this process were stored in a Google Cloud Services (GCS) bucket, from which the Derm Foundation API was used for generating the embeddings. The embeddings for each image were stored in a JSON file with the corresponding image ID as the key.

The training pipeline was built using PyTorch and was mostly common for both datasets, except for minor changes. We applied several strategies in our experimental setup to maintain reproducibility of our experiments. Firstly, we kept the seed fixed for all operations in our experiments so that all operations involving randomness, like data splitting, are consistent in different runs. The ISIC-2019 dataset was split into train (70%), validation (10%), test (20% of each class), and calibration (10% of the train set) sets with the fixed random seed, while the ASAN dataset was already pre-divided into train, test, and validation sets, and we separated 10% of the training set to form the calibration set. We also saved the model parameters from the best model state during training and loaded them later for easier usage. We maintained separate experiment logs using MLFlow for different training methodologies, which facilitated smoother experiments. We used a custom sampler as per our requirement and trained each model for 40 epochs. The custom samplers were initialized with class frequency weights, calculated as:

4 $$\begin{aligned} w_i = \frac{\frac{1}{n_i}}{\sum _{j=1}^{C}\frac{1}{n_j}} \end{aligned}$$

Where $w_i$ is the weight for class $i$, $\bar{x}_i$ is the mean F1 score from $k$ folds of cross-validation for class $i$, and $C$ is the total number of classes. As outlined previously, the sampler was updated periodically during training to adjust the data seen by the model accordingly, from the performance achieved on the validation set. During those updating processes, the following equation was used:

5 $$\begin{aligned} w_i = \frac{\frac{1}{\bar{x}_i}}{\sum _{j=1}^{k} \frac{1}{\bar{x}_j}} \end{aligned}$$

Where $w_i$ is the weight for class $i$, $n_i$ is the sample count for class $i$, and $C$ is the total number of classes. Using this training pipeline, both models were trained effectively, and the results obtained from testing with the respective test sets are discussed in the upcoming sections.

### ISIC2019 dataset

The ISIC 2019 Dataset [23] combines three notable skin cancer datasets of mostly Caucasian patients, viz., the BCN_20000 Dataset (by the Department of Dermatology, Hospital Clínic de Barcelona), the HAM10000 Dataset (by the ViDIR Group, Department of Dermatology, Medical University of Vienna), and the MSK Dataset. Along with 25,331 images of skin lesions, the ISIC 2019 dataset also contained additional patient metadata, like age, sex, general anatomical site, etc. The ISIC dataset comprises of the images, a CSV file containing the ground truth labels for each image, and another CSV file for the additional metadata. We took 23,254 embeddings, dropping the ’UNK’-labelled and the downsampled images as they were not suitable for classification. These images were distributed unevenly among 8 classes, viz. melanoma, melanocytic nevus, basal cell carcinoma, actinic keratosis, benign keratosis (solar lentigo/seborrheic keratosis), dermatofibroma, vascular lesion, and squamous cell carcinoma, with melanocytic nevus having the highest number of images (11,557) while dermatofibroma had the least (239). This was split into train (13,392), validation (3720), test (4654), and a special calibration set for the conformal prediction process (1488). The custom sampler for dynamic sampling in this training pipeline was initialized using class frequency weights as standard. Thereafter, the custom sampler was configured with F1-weights after every four epochs of training, calculated from the validation set. For the F1 weights, we performed 10-fold cross-validation and took the mean value of the classwise F1 scores for calculating the weights, which boosted the robustness of our algorithm. These weights were used for sampling with the custom sampler, with a threshold of one standard deviation above the mean of the provided weights, with a minimum baseline sampling of two standard deviations above the mean weight. Using this technique, we trained the MLP model for 40 epochs, followed by testing and producing conformal sets.

### ASAN skin cancer dataset

The ASAN Dataset was built by the Department of Dermatology at the ASAN Medical Centre by collecting clinical images of skin lesions from patients of Asian demographics and annotated by dermatologists. The ASAN skin cancer dataset was introduced by Han et al. [24] in their paper, where, alongside the ASAN dataset, they used the MED-NODE dataset and atlas site images to build a deep learning algorithm based on the Microsoft ResNet-152 model. The ASAN dataset comprises of 12,209 images of skin disorders, viz., actinic keratosis, basal cell carcinoma, dermatofibroma, hemangioma, intraepithelial carcinoma, lentigo, melanoma, melanocytic nevus, pyogenic granuloma, squamous cell carcinoma, seborrheic keratosis, and wart. Similar to the ISIC-2019 training pipeline, the ASAN pipeline was designed to dynamically sample instances from the data after each epoch based on F1- weights. The ASAN dataset comprised 12,209 images, which were divided into train (8864), validation (1086), test (1274), and calibration (985) sets. Among these classes, there is a severe imbalance, with melanocytic nevus being the largest class with 2274 instances and pyogenic granuloma being the smallest class with just 358 instances. This severe imbalance once again produced distorted results when training a classifier. We initialized the custom sampler with class frequency weights, following which the sampler uses the F1-score weights to dynamically sample class instances. The sampler weights are updated with freshly calculated F1-weights every alternate training epoch. After 40 training epochs, we test the model’s performance on the test set.

## Results

In this section, we first present the standard performance metrics for the datasets when trained individually as well as together. Next, we provide an in-depth set of results on the conformal prediction-based uncertainty quantification with respect to algorithmic fairness across patient demographics.

### Classification results

To establish a baseline performance, we have trained and tested ResNet-18 models with the datasets, the results of which are in Tables 1 and 2 for the ISIC-2019 and ASAN test sets, respectively. As observed, the baseline performance is equally matched or surpassed by the foundation model-based MLP classifier we used in our work. The added benefit of using our approach is the lesser computational complexity, training times, and ease of deployment, as expressed in Table 7. After 40 epochs of training with F1-weights using the custom sampler, the overall accuracy on the ISIC dataset on the test set was 70.33%, while the individual class metrics are highlighted in Table 3. The results when using the custom sampler and F1 weights during training (sampled columns in the table) have significantly improved minority class metrics by a 3–5% margin while maintaining unchanged performance levels for majority classes in the dataset, such as melanoma and melanocytic nevus.

**Table 1:** Classwise classification results for ISIC 2019 dataset by ResNet18 model

ISIC	Accuracy	Accuracy	F1-score	F1-score	AUC	AUC
Classes	ResNet18	MLP2048	ResNet18	MLP2048	ResNet18	MLP2048
MEL	53.73%	64.22%	0.6035	0.5992	0.62	0.77
NV	89.10%	75.43%	0.8558	0.8224	0.92	0.89
BCC	73.87%	71.28%	0.7162	0.7281	0.94	0.95
AK	40.23%	50.57%	0.4142	0.4665	0.83	0.92
BKL	47.32%	54.24%	0.4649	0.5127	0.76	0.81
DF	16.67%	72.92%	0.2500	0.5738	0.72	0.95
VASC	80.77%	94.12%	0.8750	0.8496	0.89	0.98
SCC	36.51%	70.63%	0.3898	0.4395	0.93	0.91

**Table 2:** Classwise classification results for ASAN dataset by ResNet18 model

ASAN	Accuracy	Accuracy	F1-score	F1-score	AUC	AUC
Classes	ResNet18	MLP2048	ResNet18	MLP2048	ResNet18	MLP2048
sebk	43.43%	53.54%	0.4095	0.5668	0.83	0.74
pyogenic granuloma	62.16%	89.19%	0.6765	0.5641	0.94	0.92
nevus	72.96%	82.83%	0.7203	0.8126	0.92	0.96
wart	60.61%	77.27%	0.6122	0.7445	0.90	0.94
scc	43.44%	57.83%	0.4609	0.5564	0.87	0.93
melanoma	52.54%	85.91%	0.4844	0.7759	0.92	0.90
hemangioma	65.06%	45.78%	0.5967	0.5171	0.91	0.89
intraepithelial carcinoma	36.79%	43.40%	0.4109	0.4868	0.87	0.89
lentigo	44.90%	75.51%	0.4632	0.7115	0.93	0.95
bcc	60%	72.73%	0.6111	0.6909	0.89	0.93
dermatofibroma	75%	75%	0.7250	0.7500	0.96	0.90
ak	32.26%	51.61%	0.3175	0.5120	0.85	0.90

There is a notable jump in the F1-scores of all classes due to sampling using F1-weights, which makes the model learn more from the classes on which it is facing more difficulty dynamically during training. Under the same experimental conditions, an overall accuracy of 68.83% was obtained on the ASAN dataset; the class-wise performance metrics are provided in Table 4. Using the custom sampler and F1 weights during training helps to increase metrics by 3–5% for minority classes compared to the unsampled training process, thereby balancing out performance metrics between classes. Thus, both tables show similar trends on the 2 datasets. For our combined training approach, wherein we trained a single classifier on both datasets together to develop a more robust learning model, we achieved an overall accuracy of 65.38% on the ASAN dataset and 72.49% on the ISIC2019 dataset, on the six common classes of the datasets. Detailed results are provided in Tables 5 and 6.

**Table 3:** Class-wise classification results for ISIC 2019 test set using MLP2048 model

ISIC Classes	Acc	Acc	F1-score	F1-score	Recall	Recall	AUC	AUC
	Sampled	Unsampled	Sampled	Unsampled	Sampled	Unsampled	Sampled	Unsampled
MEL	0.6422	0.6181	0.5992	0.5758	0.6422	0.6181	0.77	0.59
NV	0.7543	0.6951	0.8224	0.7914	0.7543	0.6951	0.89	0.91
BCC	0.7128	0.6286	0.7281	0.6990	0.7128	0.6286	0.95	0.96
AK	0.5057	0.5805	0.4665	0.4335	0.5057	0.5805	0.92	0.90
BKL	0.5424	0.5580	0.5127	0.4669	0.5424	0.5580	0.81	0.72
DF	0.7292	0.7500	0.5738	0.2562	0.7292	0.7500	0.95	0.89
VASC	0.9412	0.9804	0.8496	0.6757	0.9412	0.9804	0.98	0.98
SCC	0.7063	0.4365	0.4395	0.3630	0.7063	0.4365	0.91	0.89

**Table 4:** Class-wise classification results for ASAN test set with MLP2048 model

ASAN Classes	Acc	Acc	F1-score	F1-score	Recall	Recall	AUC	AUC
	Sampled	Unsampled	Sampled	Unsampled	Sampled	Unsampled	Sampled	Unsampled
ak	0.5161	0.6129	0.5120	0.5278	0.5161	0.6129	0.90	0.92
bcc	0.7273	0.6909	0.6909	0.6756	0.7273	0.7273	0.93	0.95
dermatofibroma	0.7500	0.7586	0.7500	0.7652	0.7500	0.7586	0.90	0.92
hemangioma	0.4578	0.5663	0.5171	0.5411	0.4578	0.5663	0.89	0.94
Intraepithelial carcinoma	0.4340	0.4151	0.4868	0.5057	0.4340	0.4151	0.89	0.84
lentigo	0.7551	0.7143	0.7115	0.7778	0.7551	0.7143	0.95	0.84
melanoma	0.8591	0.7455	0.7759	0.7857	0.8591	0.7455	0.90	0.95
nevus	0.8283	0.8326	0.8126	0.8308	0.8283	0.8326	0.96	0.97
Pyogenic granuloma	0.8919	0.5676	0.5641	0.5738	0.8919	0.5676	0.92	0.93
scc	0.5783	0.5492	0.5564	0.5663	0.5783	0.5492	0.93	0.96
sebk	0.5354	0.6001	0.5668	0.5742	0.5354	0.6001	0.74	0.78
wart	0.7727	0.7828	0.7445	0.7579	0.7727	0.7828	0.94	0.96

**Table 5:** Performance metrics for ISIC-2019 test set using balanced random forest classifier

ISIC	Acc	Acc	F1-Score	F1-Score	Recall	Recall	AUC	AUC
	sampled	unsampled	sampled	unsampled	sampled	unsampled	Sampled	Unsampled
AK	72.41	70.11	46.15	46.12	72.41	70.11	0.95	0.95
BCC	71.28	72.63	69.96	70.20	71.28	72.63	0.95	0.95
DF	33.33	31.25	29.09	28.04	33.33	31.25	0.94	0.94
MEL	65.42	66.87	62.63	62.78	65.42	66.87	0.84	0.85
NV	79.54	76.86	84.84	84.27	79.54	76.86	0.92	0.93
SCC	20.63	19.84	22.61	23.47	20.63	19.84	0.92	0.93

**Table 6:** Performance metrics for ASAN test set using balanced random forest classifier

ASAN	Acc	Acc	F1-Score	F1-Score	Recall	Recall	AUC	AUC
	sampled	unsampled	sampled	unsampled	sampled	unsampled	Sampled	Unsampled
ak	74.19	74.19	57.86	59.35	74.19	74.19	0.95	0.95
bcc	36.36	43.64	44.20	50.53	36.36	43.64	0.88	0.89
dermatofibroma	91.38	90.52	75.71	74.73	91.38	90.52	0.96	0.96
melanoma	59.32	59.32	67.31	66.67	59.32	59.32	0.97	0.97
nevus	64.81	65.67	73.66	75.00	64.81	65.67	0.94	0.94
scc	63.93	67.21	57.68	61.89	63.93	67.21	0.90	0.90

**Table 7:** Deployment statistics for our models

Classifier model	Params	FLOPs	Inference time
ResNet18	11 M	3.6G	162 secs
MLP2048	15.39M	30.8M	14 secs
MLP4096 with Multihead Attention	36.38M	80 M	22 secs

### Conformal set prediction

**Fig. 3:**
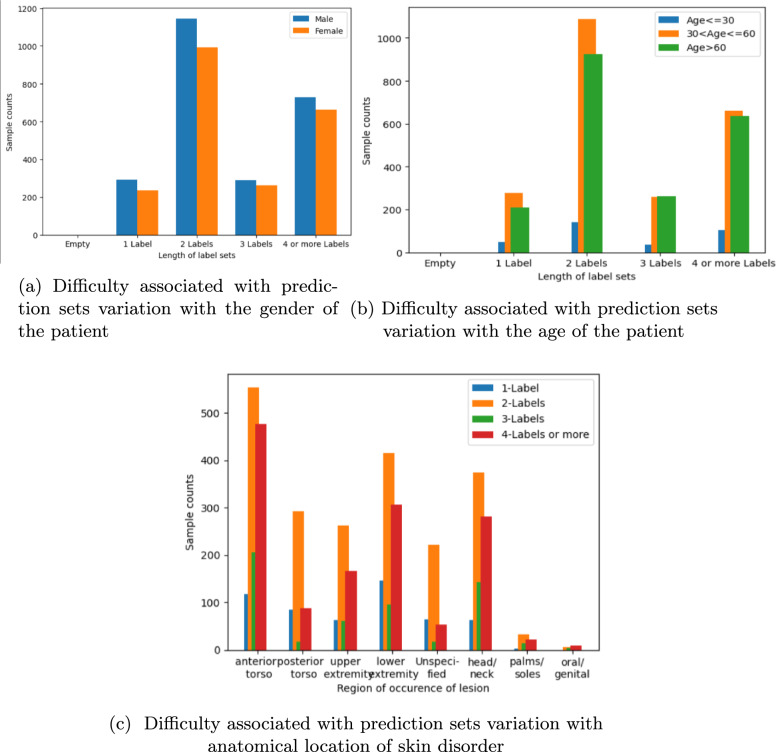
Variation of prediction set difficulty with patient metadata from ISIC2019

For conformal prediction sets with an 80% coverage guarantee, we plotted the results for the ISIC and ASAN datasets, respectively. From Fig. 3a, we can observe that the majority of the test samples have 1 or 2 labels in their prediction sets, which displays a higher confidence of the model in these test samples. A general trend observed is that skin cancer cases are observed more in male patients than in female patients. From Fig. 3b, we can observe that the majority of the test set samples have 1 or 2 labels in their conformal prediction set, with the majority of the patients being spread out in the 30–60 years age range. This output, therefore, shows that the model is producing tight confidence-bound predictions for the majority of the test samples. From Fig. 3c, we can draw an important conclusion regarding the difficulty faced by the model in generating the conformal prediction sets with respect to the anatomical site of occurrence. We observe that the anterior and posterior torso are the most common spots for skin cancer occurrence; they can be classified relatively easily, with the majority of sets containing 1 or 2 labels. For ensuring the robustness of our conformal prediction algorithm, we need to minimize the error due to calibration. Thus, we have calculated the maximum calibration error (MCE) and expected calibration error (ECE), which have extremely low values, thereby showing great performance of our conformal prediction pipeline. We have also plotted the expected vs observed coverage of the prediction sets, which coincide to show that our prediction pipeline provides the coverage guarantee it promises. The ECE and MCE values are listed in Table 8.

**Table 8:** Calibration errors for our conformal prediction pipelines

Conformal pipeline	ECE	MCE
ISIC-2019 Conformal Pipeline	0.0007848591314193063	0.0019118748249105089
ASAN Conformal Pipeline	0.00022968804256133664	0.0003599293557480232

**Fig. 4:**
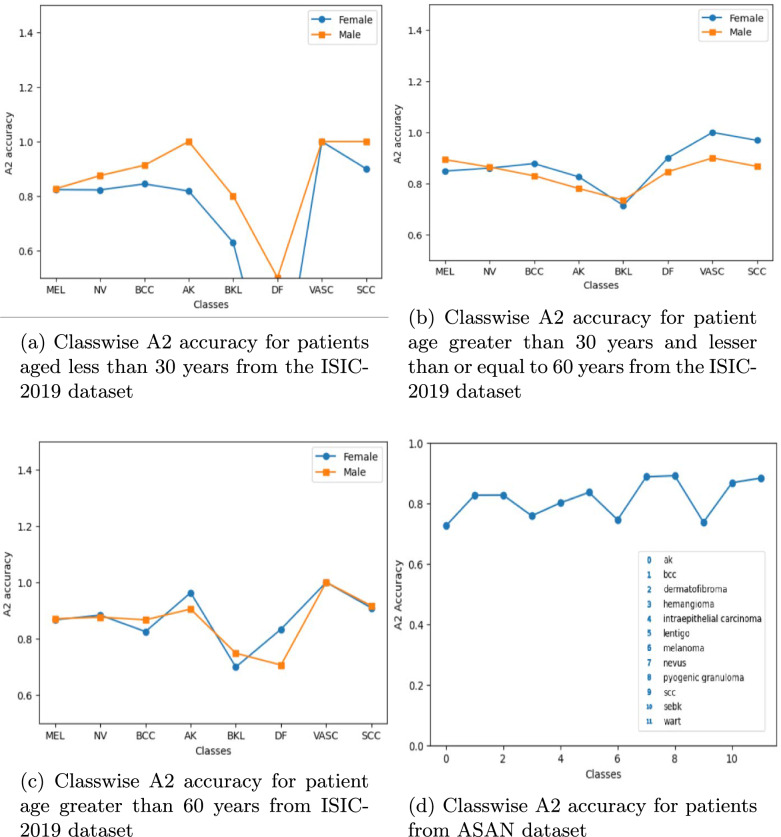
A2 accuracy for different patient categories from ISIC2019 and ASAN

For a deeper representation, we next introduce a metric —– A2 accuracy, which can be defined as the number of test samples per class that have the ground-truth label among the two most probable labels in the prediction sets, out of the total number of test samples for that class. In Fig. 4a–c, we have created a graphical representation of the classwise A2 accuracy for the ISIC dataset. Figure 4a represents statistics with patients aged below 30 years; Fig. 4b represents statistics with patients aged between 30 and 60 years; while Fig. 4c contains statistics for patients aged over 60 years. Over both male and female patients, we can observe that A2 accuracy values lie between 80 and 100% for all age ranges. Similarly, for the ASAN dataset, the A2 accuracy is shown in Fig. 4d, where we observe a similar observation hovering around the 70 to 90% range for all classes, which serves as credible proof that our model provides considerably accurate coverage within the two most probable predictions. Note that the ASAN dataset does not have the patient metadata with respect to age, gender, and anatomical sites, and hence, only one sub-figure for that dataset.

**Fig. 5:**
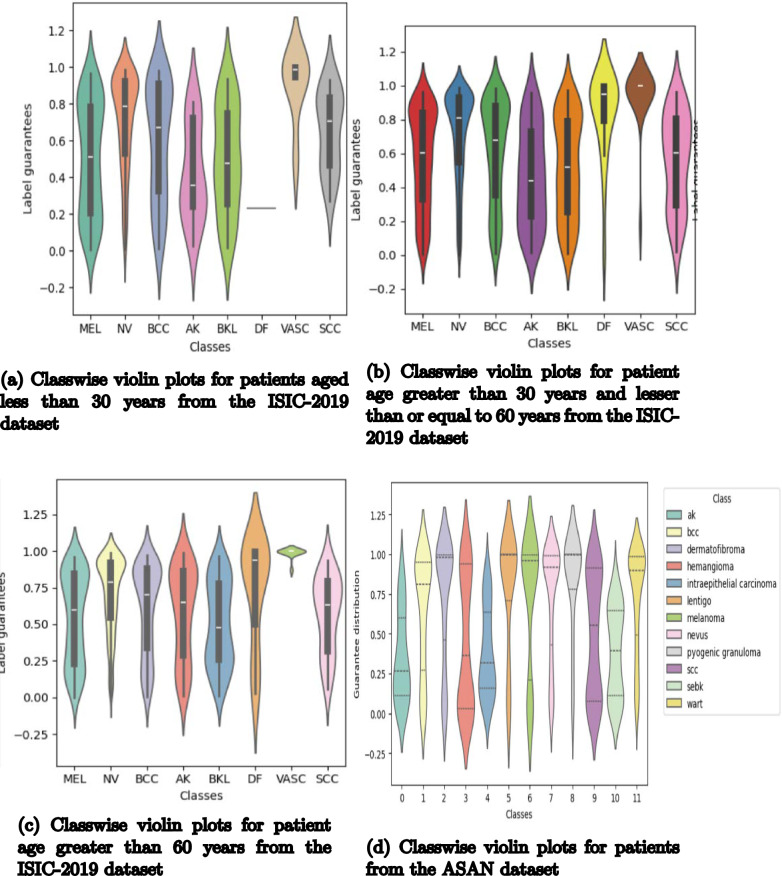
Violin plots for different patient categories from the ISIC2019 and the ASAN datasets showing ground-truth level confidences

An alternative way of visualizing the performance of the conformal prediction pipeline is to build classwise violin plots that show the distribution of the ground-truth label confidence from each set containing it as one of the possible predictions. Essentially, we should be looking out for clusters representing unimodal distributions at the upper halves of the plot. In simpler terms, this pattern would help us conclude that the model provides a guarantee in the upper half (50 to 100%) for the ground-truth labels, showing considerable confidence in the correct predictions. We can see that pattern reflected in most of the violin plots, with the mode of the distribution lying closer to one. Figure 5a–c contain three plots of classwise violin plots from the ISIC dataset, again divided by patient age; they contain patients’ data with ages lower than 30 years, between 30 and 60 years, and more than 60 years, respectively. As observed, the majority of the violin plots are skewed towards one, indicating that our model gives considerably confident predictions for the ground-truth labels for test samples. For the ASAN dataset, since most prediction sets contained multiple labels, the confidence for the ground-truth label was slightly reduced, despite being among the highest ones in that prediction set, as the total confidence coverage of 80% was distributed among multiple labels, as seen from Fig. 5d. For multiclass classification problems with complex features and a larger number of classes, this could be a potential issue. For the majority classes, like melanoma and melanocytic nevus, due to the large number of test samples, the plots are denser when compared to minority classes like dermatofibroma and vascular lesion. For these scatter plots, our target is to have sparse points towards zero, which would indicate that the ground-truth confidence lies among the top two predictions in the set and has either a majority or considerable guarantee (and might require examination from a human expert to make the final decision).

**Fig. 6:**
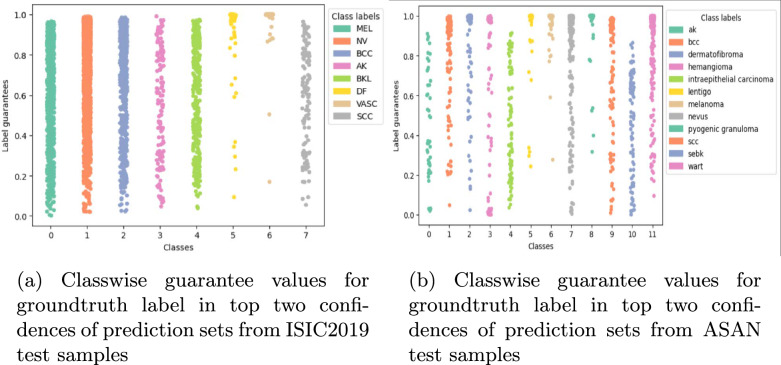
Classwise guarantee values for groundtruth label in top two confidences of prediction sets from test samples

An effective strategy for tackling the problem of low confidence due to multiple labels can be developed by combining the strategies of the A2 accuracy and ground-truth guarantee, by observing the ground-truth guarantee if it is present in the top two guarantees of the prediction set. Figure 6a shows the scatter plot for such ground-truth guarantees, if present among the top two labels of respective prediction sets for the ISIC dataset. All the classwise scatter plots in Fig. 6a have concentrated clusters towards the higher confidence regions, and some scattered points nearer to zero. Utilising the additional metadata for the ISIC dataset, we also recorded the most common anatomical region of occurrences for skin disorders where the ground truth lies among the top two predictions in the prediction set. This data can be of great use when using the model for diagnosis applications, as a high guarantee towards a particular class for a test sample, especially in one of the more commonly occurring anatomical regions, can be safely considered as a correct diagnosis. This data is summarized in Table 10, where the most common anatomical region of occurrences is listed in decreasing order of occurrence. For the ASAN dataset, we created a similar scatter plot with ground-truth prediction among the top two predictions of the set, as shown in Fig. 6b. All the classwise scatter plots have dense clusters towards the higher guarantee regions and only some scarce points around zero. Thus, conformal prediction-based uncertainty quantification, when presented in different ways, teases out a lot of valuable information regarding robustness and fairness of performance across different patient groupings, thus serving as a generalised metric for algorithmic fairness.

We also decided to include some additional results on individual samples that directly showcase the power of conformal prediction. As we can see, in Table 9, among the 4 examples from the ISIC-2019 dataset, there are 4 distinct scenarios. In the first case (ISIC_0031240), our method (with a novel F1-based adaptive sampling strategy) provides the correct classification (NEV) while the base pipeline (without adaptive sampling) misclassifies as MEL, and also the confidence is high since the conformal set only has 2 entries, with the top entry being NEV with 85.44%. For the second case (ISIC_0026992), again, our method provides the correct classification label (SCC) while the compared method does not (DF); however, you can see from the conformal set that the confidence is lower, as this time there should be 3 entries to meet the coverage level, and the top entry SCC only has 55.36%. Thus, the varying percentage and length of the conformal set becomes a measure of individualised predictive uncertainty for a particular test sample, as a measure of how challenging it is to classify.

Next, we present two misclassification examples. For the third case (ISIC_0061690), we see that the baseline model got the correct label, SCC, while our model did not (BCC). However, the conformal set still contains the correct label as the second entry with a similar percentage (SCC, 42.53%) as the first entry of the wrong label (BCC, 43.88%). This highlights a great benefit of conformal prediction, as this provides the human expert looking at the results to consider SCC as a potential candidate even though the top (wrong) result is BCC for this particular sample. Finally, in the fourth and last scenario, both models get it wrong (BCC instead of DF), and indeed, the conformal set also does not contain the ground truth label.

We have included the sample images within Table 9. We hope that this provides any reader with a clearer intuition of the benefits and motivation of conformal prediction-based uncertainty quantification.

**Table 9:** Comparison of ground truth, pipeline prediction, and baseline prediction with conformal confidence sets for some random samples

Test Sample	Ground Truth (GT) Class	Prediction by our Pipeline	Prediction by Baseline	Conformal Set (GT in bold)
ISIC_0031240	NEV	NEV	MEL	{ **‘NEV’**: 85.44%, ‘MEL’: 05.74% }
ISIC_0026992	SCC	SCC	DF	{ **‘SCC’**: 55.36%, ‘DF’: 34.67%, ‘BCC’: 03.33% }
ISIC_0061690	SCC	BCC	SCC	{ ‘BCC’: 43.88%, **‘SCC’**: 42.53%, ‘AK’: 12.56% }
ISIC_0055258	DF	BCC	BCC	{ ‘BCC’: 55.28%, ‘SCC’: 21.05%, ‘AK’: 20.67% }

**Table 10:** Classwise distribution of the most common region of occurrences

Class	MEL	NV	BCC	AK	BKL	DF	VASC	SCC
Region 1	Lower	Anterior	Anterior	Anterior	Anterior	Anterior	Anterior	Anterior
	extremity (27.34%)	torso (25.15%)	torso (41.12%)	torso (42.00%)	torso (39.94%)	torso (39.47%)	torso (40.00%)	torso (38.79%)
Region 2	Posterior	Lower	head/	head/	head/	head/	head/	head/
	torso (21.76%)	extremity (19.45%)	neck (25.13%)	neck (19.66%)	neck (27.24%)	neck (18.42%)	neck (22.00%)	neck (22.41%)
Region 3	Anterior	Anterior	Upper	Lower	head/	head/	Lower	Lower
	torso (13.67%)	torso (17.70%)	extremity (18.80%)	extremity (16.88%)	neck (19.20%)	neck (18.42%)	extremity (22.00%)	extremity (20.69%)
Region 4	nan	Posterior	Upper	Upper	Upper	Upper	Upper	Upper
	(13.53%)	torso (13.05%)	extremity (10.37%)	extremity (12.67%)	extremity (9.29%)	extremity (10.53%)	extremity (16.00%)	extremity (15.22%)
Region 5	Upper	Upper	palms/	nan	nan	palms/		palms/
	extremity (13.11%)	extremity (12.50%)	soles (2.64%)	(2.00%)	(2.79%)	soles (5.26%)		soles (1.72%)
Region 6	head/	nan	nan	palms/	palms/	nan		nan
	neck (10.60%)	(10.15%)	(1.05%)	soles (1.33%)	soles (1.24%)	(5.26%)		(0.86%)
Region 7		palms/	oral/	oral/	oral/	oral/		
		soles (1.65%)	genital (0.88%)	genital (0.67%)	genital(0.31%)	genital(2.63%)		
Region 8		oral/						
		genital(0.35%)						

## Conclusion

In this work, we have demonstrated that conformal prediction-based uncertainty quantification can function as a powerful metric for algorithmic fairness and robustness, which provides a coverage guarantee at a user-specified level of significance that the true prediction is contained within the ‘conformal set’. This adds a level of trustworthiness to the AI pipeline towards adoption in high-risk applications like healthcare. We have chosen skin lesion classification as the predictive task in this paper because it provides a strong exemplar of class imbalance due to the overwhelming ethnic bias in favour of Caucasian patients. We have introduced a novel dynamic sampling strategy that uses F1 scores during training to select samples judiciously across challenging classes and hence ends up with a more equitable performance across patient demographics.

The proposed predictive pipeline, including the conformal prediction layer, paired with the fairness in classification between majority and minority classes, should provide a robust framework for preliminary screenings for suspected skin cancer patients. The balanced classifiers should work equivalently for all the skin cancer categories, and if the label set contains any one skin cancer subtype with high probability, then the diagnosis process of that subtype can be carried out on the patient; meanwhile, if multiple subtypes all coexist with significant probabilities, then detailed diagnosis by skilled clinicians becomes necessary, thus making the overall screening process much safer and trustable. This system could also provide services in remote locations with limited clinician access, wherein complicated cases can be referred for further diagnosis. Thus, this skin cancer prediction system, along with the conformal prediction pipeline, can help clinicians reduce their effort considerably while diagnosing cases.

Both the conformal prediction and the F1 dynamic sampler are task and model-agnostic frameworks that can be generalised to other similar tasks and datasets. Despite the deluge of papers being published in health AI, very few of them get deployed to the clinic, and this clinical translation bottleneck will only get exacerbated with emerging legislation around the world regarding AI safety in high-risk applications like healthcare. In such a scenario, a simple yet rigorous approach like conformal prediction can help AI developers to add a layer of trustworthiness to their model without having to compromise on the deep learning architecture itself. Since our method provides a conformal set for each individual patient, it can also be a progressive step towards the grand challenge of personalised healthcare and precision medicine.

### Limitations and future work

 Despite our approach of trying to comprehensively cover multiple real-world situations, the system has certain limitations. Since the classifier is trained on embeddings from different subtypes of malignant skin lesions, benign skin lesions might not be properly classified by the system. Since the dermatological images were captured directly from patient skin patches, distorted or obstructed images might lead to erroneous predictions. The immediate next phase of work would be to achieve clinical validation, both through benchmarking on an expanded real-world dataset as well as through feedback from dermatologists and clinical experts. Thereafter, further technical advancements might be explored, like learning predictive morphological features as clinically established concepts and expanding the conformal framework to encapsulate these for further explainability and trustworthiness. Finally, more future-oriented work might include incorporating multimodal data like histopathology and clinicogenomics into an integrated pipeline.

## Data Availability

All data used in this work are taken from public open access benchmark datasets, ISIC (https://www.kaggle.com/datasets/salviohexia/isic-2019-skin-lesion-images-for-classification) and (https://figshare.com/articles/figure/Asan_and_Hallym_Dataset_Thumbnails_/5406136). The datasets are all anonymised with prior ethics in place as part of the original studies from which they were generated, no new data was generated as part of this work.

## Footnotes

1 https://seer.cancer.gov/statfacts/html/melan.html
